# An enigma of hypothyroidism and hyponatremia coexistence: a nationwide population-based retrospective study

**DOI:** 10.1186/s12889-023-16791-5

**Published:** 2023-09-29

**Authors:** Chun-Hao Chu, Wu-Chien Chien, Chiung-Chen Liu, Chi-Hsiang Chung, Ying-Chuan Chen, Feng-Chih Kuo, Hung-Hsiang Fang, Cheng-Yi Cheng, Yi-Xuan Ding, Chiung-Hsi Tien, Chien-Ming Lin

**Affiliations:** 1 Department of Pediatrics, Zuoying Branch of Kaohsiung Armed Forces General Hospital, Kaohsiung, Taiwan; 2 Department of Pediatrics, Tri-Service General Hospital, National Defense Medical Center, Taipei, Taiwan; 3 School of Public Health, National Defense Medical Center, Taipei, Taiwan; 4 Department of Medical Research, Tri-Service General Hospital, National Defense Medical Center, Taipei, Taiwan; 5 Graduate Institute of Life Sciences, National Defense Medical Center, Taipei, Taiwan; 6 Taiwanese Injury Prevention and Safety Promotion Association, Taipei, Taiwan; 7 Department of Physiology & Biophysics, National Defense Medical Center, Taipei, Taiwan; 8 Division of Endocrinology and Metabolism, Department of Internal Medicine, Tri-Service General Hospital, National Defense Medical Center, Taipei, Taiwan; 9 PET Center and Department of Nuclear Medicine, Tri-Service General Hospital, National Defense Medical Center, Taipei, Taiwan; 10 School of Medicine, National Defense Medical Center, Taipei, Taiwan

**Keywords:** Hypothyroidism, Hyponatremia, Mortality, Length of stay

## Abstract

**Introduction:**

Hypothyroidism is a rare and possible cause of hyponatremia. However, the clinical epidemiology and risk of mortality (ROM) when they coexist still remain elusive.

**Objectives:**

We assessed the epidemiology and ROM among index patients with coexisting hypothyroidism and hyponatremia via a national population database.

**Patients and methods:**

This retrospective cohort study utilized Taiwan’s National Health Insurance program database. Distributions of definite sociodemographic factors were analyzed. The annual incidence among the overall group and sex-subgroups was investigated. In addition, potential factors influencing the ROM were also evaluated.

**Results:**

Of 4,549,226 patients from 1998 to 2015, a total of 3,140 index patients with concurrent hypothyroidism and hyponatremia were analyzed. The incidence rate increased tenfold from 1998 to 2015; average annual incidence rate was 174. Among the total participants, 57.1% were women; mean age was 72.6 ± 14.7 years and 88.8% were aged > 55 years. Although average length of stay (LOS) was 13.1 ± 15.4 days, the mortality group had significantly longer LOS than that in the survival group (12.9 days vs 22.2 days). Old age, catastrophic illness, cardiac dysrhythmia, and low hospital hierarchy were independent predictors of hospital mortality. The optimal LOS cutoff value for ROM prediction was 16 days. Index patients with LOS > 16 days increased ROM by 2.3-fold.

**Conclusions:**

Coexistent hypothyroidism and hyponatremia is rare, although the incidence increased gradually. Factors influencing the ROM, such as old age, underlying catastrophic status, cardiac dysrhythmia, hospital hierarchy, and LOS should be considered in clinical care.

**Supplementary Information:**

The online version contains supplementary material available at 10.1186/s12889-023-16791-5.

## Introduction

Hypothyroidism has been reported to be associated with severe hyponatremia, which can contribute to a high mortality rate [1, 2]. The main mechanisms of hypothyroidism causing profound hyponatremia included decreased cardiac output and reduced capacity of free water excretion due to increased antidiuretic hormone (ADH) levels [3–5]. A recent study found that hypothyroidism-induced hyponatremia is rather rare and probably occurs only in patients with severe hypothyroidism and myxedema coma [3]; however, distinct electrolyte dysregulation poses the risk of permanent or fatal neurologic sequelae in hospitalized patients. Thus, a comprehensive investigation of the epidemiology of coexistent hypothyroidism and hyponatremia is extremely important, not only to increase knowledge of this rare nephron-endocrinological disease, but also to investigate mortality predictors in such patients.

Patients with hyponatremia have a high risk of readmission and increased length of hospital stay (LOS), which emphasizes the clinical importance and public health impact of hyponatremia [6]. In addition, acute severe hyponatremia might contribute to serious neurological consequences due to cerebral edema, and it could be lethal if not recognized early as a concurrent curable disease [7]. The direct costs of treating hyponatremia in the USA range between $1.6 billion and $3.6 billion annually; [8] hospital costs per patient are $3000 higher in patients with hyponatremia than in those with normonatremia [9]. Although it is well known that the treatment of hyponatremia represents a significant healthcare burden worldwide, epidemiological studies of coexistent hypothyroidism are limited, especially in Asia. Crucially, prompt recognition of the risk of mortality (ROM) that might decrease the medical costs of hyponatremia in the inpatient setting could also minimize the burden associated with the concomitant disease. [8, 10–12]

To explicitly unravel this issue, we conducted a nationwide population-based study which aimed to comprehensively assess the epidemiology and ROM among patients with coexisting hypothyroidism and hyponatremia.

## Methods

### Data sources

The National Health Insurance (NHI) program began in Taiwan in 1995 and covers more than 99% of the entire population, with approximately 23 million beneficiaries [13]. This retrospective cohort study was conducted using inpatient files from the Taiwan NHI Research Database (NHIRD). Diagnostic codes based on the ICD-9-CM were retrieved from NHIRD [13, 14]. The ICD-9-CM codes of interested analytic diagnoses were included in the study group, such as hypo-osmolality and/or hyponatremia (ICD-9-CM 276.1) and unspecified acquired hypothyroidism (ICD-9-CM 244.9). In addition, patients with either known or newly diagnosed hypothyroidism were both eligible to be enrolled in this study. Other detailed information of the ICD-9-CM codes used in this study is provided in eTable 1 in the Supplement.

### Ethical considerations

The NHIRD encrypts patients’ personal information for privacy and provides researchers with anonymous identification numbers associated with relevant claim information, including patients’ sex, dates of birth, medical services utilized, and prescriptions. Since all identifying personal information was stripped from the secondary files before analysis, patient consent was not required for accessing the NHIRD. The Ethics Committee of the Institutional Review Board of Tri-Service General Hospital, National Defense Medical Center approved this study (TSGHIRB No. B-111–17).

### Study design and population

Inpatients simultaneously diagnosed with hyponatremia and hypothyroidism from January 1, 1998 to December 31, 2015 were selected from the NHIRD. Data from the “detailed documents of hospitalization medical expenses” and “registry for contracted medical facilities” were extracted from the NHIRD. The index date was defined as the date when the patients were first diagnosed with hypothyroidism or hyponatremia. The exclusion criteria were as follows: (1) hypothyroidism before the index date; (2) hyponatremia before tracking; (3) hyponatremia but without hypothyroidism and vice-versa; (4) unknown age; (5) unknown sex.

### Covariates

We examined sociodemographic factors, including age, monthly income, season, location of residence, urbanization level, and hospital level. The monthly income in terms of New Taiwan Dollars (NTD) was divided into two groups, namely < 18,000 (low-income) and ≥ 18,000 (not low-income). The impact of four seasons (spring, summer, autumn, and winter) on the epidemiology of hyponatremia and/or hypothyroidism was investigated. Patients living in different areas of Taiwan, including northern, middle, southern, and eastern Taiwan, as well as the outlet islands, were compared. The patients’ habitats were categorized into four urbanization levels from the highest (1) to the lowest (4) according to the population of the region (level 1: population more than 1,250,000; level 2: population more than 500,000 but less than 1,250,000; level 3: population more than 100,000 but less than 500,000; level 4: population less than 100,000). Three levels for hospitals where the patients sought medical attention were considered, namely medical centers, regional hospitals, and local hospitals.

### Comorbidities

The baseline comorbidity history included diabetes mellitus (DM; ICD‐9‐CM 250), disorders of adrenal glands (ICD‐9‐CM 255), endocrine dysfunction (ICD‐9‐CM 258.1), hypertension (HTN; ICD‐9‐CM 401–405), ischemic heart disease (IHD; ICD‐9‐CM 410–414), heart failure (HF; ICD‐9‐CM 428), chronic kidney disease (CKD; ICD‐9‐CM 585), chronic obstructive pulmonary disease (COPD; ICD‐9‐CM 490–496), asthma (ICD‐9‐CM 493), mental disorders (ICD‐9‐CM 290–319), diseases of the nervous system (ICD‐9‐CM 320–389), cardiac dysrhythmias (ICD‐9‐CM 427), benign prostatic hypertrophy (BPH; ICD‐9‐CM 600.00), cardiomyopathy (ICD‐9‐CM 425), sickle-cell disease (ICD‐9‐CM 282.60), and liver cirrhosis (ICD‐9‐CM 571). These comorbidities were included in the models as categorical variables.

### Main outcome measures

All participants with hypothyroidism were followed from the index date until the onset of hyponatremia, as recorded in the NHIRD. Distributions of definite sociodemographic factors, including age, residential geographic area, inpatient season, catastrophic illness (with, without), and low-income household (with, without) were analyzed. The annual incidence for the overall group and sex-subgroups was investigated. In addition, the potential factors influencing the ROM were thoroughly evaluated among survival and mortality groups.

### Statistical analyses

The chi-square test was used to analyze differences between the categorical variables. Fisher’s exact test was used to evaluate differences between the survival and mortality groups. Continuous variables were compared using a one-way analysis of variance. Logistic regression model analysis was used to assess the odds ratio (OR) of mortality in index patients after adjusting for appropriate covariates, and the data were expressed as adjusted OR with a 95% confidence interval (CI). All statistical analyses were performed using SPSS software v.22.0 (SPSS, Chicago, IL), and *P* < 0.05 was considered to be statistically significant.

## Results

### Study population

Among the 4,549,226 hospitalized patients (eFigure 1), we identified 354,134 patients with a diagnosis of hypo-osmolality/hyponatremia from 1998 to 2015 in Taiwan (eTable 2). Among them, 3,142 study participants (0.89%) were simultaneously diagnosed to have developed hypothyroidism. After excluding patients with unknown age and sex, 3,140 index patients were finally analyzed (eFigure 1). During the period of 18 years, the average incidence was 174 affected patients per year.

### Demographic characteristics of the study population

In patients with coexisting hypothyroidism and hyponatremia, the proportion of females was greater than that of males (57.1% vs 42.8%). The mean age of study participants was 72.6 ± 14.7 years, and the proportion of patients aged > 55 years was 88.9% (Table 1). The proportion of patients from low-income households was 2.4%. The proportion of patients with a catastrophic illness was 15.3% [15]. Although no difference in the hospitalization rate was noted among the four seasons, a higher proportion of patients were living in northern Taiwan (39.9%) and the city (76.1%, levels 1 and 2). Regarding the medical care system, a higher proportion of patients sought medical treatment at a regional hospital (45.8%). Patients with surgery accounted for 4.27%, the average hospital LOS was 13.1 ± 15.4 days, and medical expenses were 58 211 NTD on average.

**Table 1:** Characteristics of index patients with coexistent hypothyroidism and hyponatremia

Characteristics	Total		Survive		Mortality		*P*-value
	n	%	n	%	n	%	
**Overall**	3,140		3,069	97.74	71	2.26	
**Gender**							0.717
Male	1,346	42.87	1,314	42.82	32	45.07	
Female	1,794	57.13	1,755	57.18	39	54.93	
**Age (yrs)**	72.58 ± 14.73		72.42 ± 14.77		79.71 ± 10.48		**<0.001**
**Age group (yrs)**							**0.013**
0–4	9	0.29	9	0.29	0	0.00	
5–14	13	0.41	13	0.42	0	0.00	
15–24	13	0.41	13	0.42	0	0.00	
25–34	44	1.40	44	1.43	0	0.00	
35–44	66	2.10	66	2.15	0	0.00	
45–54	205	6.53	204	6.65	1	1.41	
55–64	417	13.28	411	13.39	6	8.45	
65–74	731	23.28	717	23.36	14	19.72	
75–84	1,093	34.81	1,068	34.80	25	35.21	
≧85	549	17.48	524	17.07	25	35.21	
**Low-income**							0.575
Without	3,064	97.58	2,994	97.56	70	98.59	
With	76	2.42	75	2.44	1	1.41	
**Catastrophic illness**							**<0.001**
Without	2,660	84.71	2,613	85.14	47	66.20	
With	480	15.29	456	14.86	24	33.80	
**DM**							0.515
Without	2,628	83.69	2,566	83.61	62	87.32	
With	512	16.31	503	16.39	9	12.68	
**Disorders of adrenal glands**							0.225
Without	2,699	85.96	2,634	85.83	65	91.55	
With	441	14.04	435	14.17	6	8.45	
**Endocrine dysfunction**							0.879
Without	3,139	99.97	3,068	99.97	71	100.00	
With	1	0.03	1	0.03	0	0.00	
**HTN**							**<0.001**
Without	2,513	80.03	2,445	79.67	68	95.77	
With	627	19.97	624	20.33	3	4.23	
**IHD**							0.802
Without	2,943	93.73	2,877	93.74	66	92.96	
With	197	6.27	192	6.26	5	7.04	
**HF**							0.068
Without	2,955	94.11	2,892	94.23	63	88.73	
With	185	5.89	177	5.77	8	11.27	
**CKD**							0.555
Without	3,001	95.57	2,934	95.60	67	94.37	
With	139	4.43	135	4.40	4	5.63	
**COPD**							0.376
Without	2,883	91.82	2,820	91.89	63	88.73	
With	257	8.18	249	8.11	8	11.27	
**Asthma**							0.395
Without	3,109	99.01	3,038	98.99	71	100.00	
With	31	0.99	31	1.01	0	0.00	
**Mental disorders**							0.321
Without	2,945	93.79	2,876	93.71	69	97.18	
With	195	6.21	193	6.29	2	2.82	
**Disorder of thyroid**							-
Without	3,140	100.00	3,069	100.00	71	100.00	
With	0	0.00	0	0.00	0	0.00	
**Diseases of the nervous system**							0.796
Without	2,755	87.74	2,692	87.72	63	88.73	
With	385	12.26	377	12.28	8	11.27	
**Cardiac dysrhythmias**							**0.022**
Without	2,985	95.06	2,922	95.21	63	88.73	
With	155	4.94	147	4.79	8	11.27	
**BPH**							0.625
Without	3,097	98.63	3,026	98.60	71	100.00	
With	43	1.37	43	1.40	0	0.00	
**Cardiomyopathy**							0.761
Without	3,136	99.87	3,065	99.87	71	100.00	
With	4	0.13	4	0.13	0	0.00	
**Sickle-cell disease**							-
Without	3,140	100.00	3,069	100.00	71	100.00	
With	0	0.00	0	0.00	0	0.00	
**Liver cirrhosis**							0.627
Without	3,092	98.47	3,021	98.44	71	100.00	
With	48	1.53	48	1.56	0	0.00	
**Operations on bone marrow and spleen**							0.792
Without	3,137	99.90	3,066	99.90	71	100.00	
With	3	0.10	3	0.10	0	0.00	
**Season**							0.306
Spring (Mar-May)	729	23.22	713	23.23	16	22.54	
Summer (Jun-Aug)	762	24.27	745	24.28	17	23.94	
Autumn (Sep-Nov)	812	25.86	799	26.03	13	18.31	
Winter (Dec-Feb)	837	26.66	812	26.46	25	35.21	
**Location**							0.448
Northern Taiwan	1,254	39.94	1,227	39.98	27	38.03	
Middle Taiwan	785	25.00	764	24.89	21	29.58	
Southern Taiwan	937	29.84	916	29.85	21	29.58	
Eastern Taiwan	149	4.75	148	4.82	1	1.41	
Outlets islands	15	0.48	14	0.46	1	1.41	
**Urbanization level**							0.184
1 (The highest)	986	31.40	969	31.57	17	23.94	
2	1,403	44.68	1,372	44.71	31	43.66	
3	199	6.34	195	6.35	4	5.63	
4 (The lowest)	552	17.58	533	17.37	19	26.76	
**Level of care**							**<0.001**
Hospital center	1,164	37.07	1,148	37.41	16	22.54	
Regional hospital	1,437	45.76	1,407	45.85	30	42.25	
Local hospital	539	17.17	514	16.75	25	35.21	
**Surgery**							0.769
Without	3,006	95.73	2,937	95.70	69	97.18	
With	134	4.27	132	4.30	2	2.82	
**Length of stay (days)**	13.14 ± 15.41		12.93 ± 14.52		22.25 ± 36.24		**<0.001**
**Length of days groups**							**0.010**
≦30	2,899	92.32	2,840	92.54	59	83.10	
> 30	241	7.68	229	7.46	12	16.90	
**Medical cost (NT$)**	58,211.36 ± 91,002.51		56,972.80 ± 88,847.38		111,748.15 ± 149,710.89		**<0.001**
**Repeated inpatient**							0.370
Without	2,731	86.97	2,672	87.06	59	83.10	
With	409	13.03	397	12.94	12	16.90	

*p*-value (categorical variable: Chi-square test/Fisher’s exact test; continuous variable: t-test)

### Demographic characteristics between survival and mortality subgroups

The index patients were classified into survival (n = 3,069) and mortality (*n* = 71) subgroups (Table 1). In particular, there was a significant difference in age between these two groups (72.4 years vs 79.1 years; *P* < 0.001). Moreover, patients aged < 45 years were not observed in the mortality group. The proportion of those experiencing catastrophic illness was significantly higher in the mortality group (14.9% vs 33.8%). Although the proportion of the survival group having HTN (20.3%) was significantly higher than that of the mortality group, the latter had an increased proportion of cardiac dysrhythmia (4.8% vs 11.3%; *P* = 0.022). Considering the hospital hierarchy, the proportion of patients seeking medical treatment at a local hospital (lowest level) was higher in the mortality group (16.8% vs 35.2%, *P* < 0.001). Compared to the survival group, the mortality group had a significantly longer LOS (12.9 days vs 22.2 days; *P* < 0.001) and the distinct proportions of LOS > 30 days (7.5% vs 16.9%, *P* = 0.010). In addition, the mortality group had significantly higher expenditures (56,972 NTD vs 111,748 NTD; *P* < 0.001).

### Risk of mortality in index patients stratified by covariates

Patients aged ≥ 85 years had a higher ROM than those aged 45–84 years after adjusting for the variables listed in Table 2 (all *P* < 0.05), indicating that old age might be a contributory factor in the mortality of patients with coexisting hypothyroidism and hyponatremia. In addition, the ROM due to catastrophic illness was 3.9 times more than that of non-catastrophic illness (*P* < 0.001). Notably, the index patients with underlying HTN depicted decreased ROM (0.2-fold, *P* = 0.007), but those with cardiac dysrhythmia showed 2.1 times increased ROM than those without (*P* = 0.078). After adjusting for variables, comorbidities such as DM, disorders of adrenal glands, endocrine dysfunction, IHD, HF, CKD, COPD, asthma, mental disorders, diseases of the nervous system, BPH, cardiomyopathy, sickle-cell disease, and liver cirrhosis did not contribute to the ROM.

**Table 2:** Logistic regression analysis of factors influencing mortality among index patients

Variables	Crude OR	95% CI	95% CI	*P*-value	Adjusted OR	95% CI	95% CI	*P*-value
**Gender**								
Male	1.096	0.683	1.759	0.704	0.908	0.547	1.508	0.709
Female	Reference				Reference			
**Age group (yrs)**								
0–4	0.000	-	-	0.999	0.000	-	-	0.999
5–14	0.000	-	-	0.999	0.000	-	-	0.999
15–24	0.000	-	-	0.999	0.000	-	-	0.999
25–34	0.000	-	-	0.998	0.000	-	-	0.997
35–44	0.000	-	-	0.997	0.000	-	-	0.997
45–54	0.103	0.014	0.763	**0.026**	0.074	0.010	0.576	**0.013**
55–64	0.306	0.124	0.753	**0.010**	0.231	0.088	0.606	**0.003**
65–74	0.409	0.211	0.795	**0.008**	0.356	0.175	0.726	**0.005**
75–84	0.491	0.279	0.863	**0.013**	0.445	0.246	0.803	**0.007**
≧85	Reference				Reference			
**Low-income**								300
Without	Reference				Reference			
With	0.570	0.078	4.160	0.580	0.821	0.101	6.637	0.853
**Catastrophic illness**								
Without	Reference				Reference			
With	2.926	1.772	4.832	** <0.001**	3.912	2.170	7.052	**<0.001**
**DM**								
Without	Reference				Reference			
With	0.741	0.366	1.500	0.404	0.989	0.472	2.073	0.977
**Disorders of adrenal glands**								
Without	Reference				Reference			
With	0.559	0.241	1.298	0.176	0.865	0.359	2.085	0.746
**Endocrine dysfunction**								
Without	Reference				Reference			
With	0.000	-	-	0.999	0.000	-	-	0.999
**HTN**								
Without	Reference				Reference			
With	0.173	0.054	0.551	**0.003**	0.198	0.061	0.640	**0.007**
**IHD**								
Without	Reference				Reference			
With	1.135	0.452	2.851	0.787	1.029	0.395	2.679	0.953
**HF**								
Without	Reference				Reference			
With	2.075	0.979	4.397	0.057	1.620	0.733	3.581	0.233
**CKD**								
Without	Reference				Reference			
With	1.298	0.466	3.611	0.618	1.438	0.495	4.181	0.505
**COPD**								
Without	Reference				Reference			
With	1.438	0.681	3.035	0.340	1.041	0.469	2.307	0.922
**Asthma**								
Without	Reference				Reference			
With	0.000	-	-	0.998	0.000	-	-	0.998
**Mental disorders**								
Without	Reference				Reference			
With	0.432	0.105	1.775	0.244	0.453	0.107	1.914	0.281
**Diseases of the nervous system**								
Without	Reference				Reference			
With	0.907	0.431	1.907	0.796	1.054	0.486	2.286	0.893
**Cardiac dysrhythmias**								
Without	Reference				Reference			
With	2.524	1.187	5.365	**0.016**	2.066	0.921	4.636	0.078
**BPH**								
Without	Reference				Reference			
With	0.000	-	-	0.998	0.000	-	-	0.998
**Cardiomyopathy**								
Without	Reference				Reference			
With	0.000	-	-	0.999	0.000	-	-	0.999
**Sickle-cell disease**								
Without	Reference				Reference			
With	-	-	-	-	-	-	-	-
**Liver cirrhosis**								
Without	Reference				Reference			
With	0.000	-	-	0.998	0.000	-	-	0.997
**Operations on bone marrow and spleen**								
Without	Reference				Reference			
With	0.000	-	-	0.999	0.000	-	-	0.999
**Season**								
Spring (Mar-May)	Reference				Reference			
Summer (Jun-Aug)	1.017	0.510	2.028	0.962	0.925	0.453	1.890	0.831
Autumn (Sep-Nov)	0.725	0.346	1.518	0.394	0.694	0.324	1.487	0.347
Winter (Dec-Feb)	1.372	0.727	2.590	0.329	1.409	0.730	2.719	0.307
**Location**					**Multicollinearity with urbanization level**			
Northern Taiwan	Reference				**Multicollinearity with urbanization level**			
Middle Taiwan	1.249	0.701	2.225	0.450	**Multicollinearity with urbanization level**			
Southern Taiwan	1.042	0.585	1.855	0.889	**Multicollinearity with urbanization level**			
Eastern Taiwan	0.307	0.041	2.276	0.248	**Multicollinearity with urbanization level**			
Outlets islands	3.246	0.412	25.578	0.264	**Multicollinearity with urbanization level**			
**Urbanization level**								
1 (The highest)	0.492	0.254	0.955	**0.036**	0.739	0.341	1.604	0.445
2	0.634	0.355	1.132	0.123	0.848	0.452	1.593	0.609
3	0.575	0.193	1.713	0.321	0.689	0.224	2.124	0.517
4 (The lowest)	Reference				Reference			
**Level of care**								
Medical center	0.287	0.152	0.541	** <0.001**	0.344	0.162	0.734	**0.006**
Regional hospital	0.438	0.255	0.752	**0.003**	0.529	0.295	0.948	**0.032**
Local hospital	Reference				Reference			
**Surgery**								
Without	Reference				Reference			
With	0.645	0.156	2.659	0.544	0.766	0.177	3.310	0.721
**Length of stay (days)**	1.018	1.010	1.026	** <0.001**	1.009	0.998	1.020	0.099
**Medical cost (NT$)**	1.000	1.000	1.000	**0.001**	**Multicollinearity with length of days**			
**Repeated inpatient**								
Without	Reference				Reference			
With	1.369	0.729	2.569	0.328	0.980	0.452	2.127	0.959

Adjusted OR: Adjusted Odds Ratio; *CI* confidence interval

Adjusted OR: adjusted all variables listed in the above table

Nagelkerke R-square of adjusted model = 0.160

Location of inpatients had multicollinearity with urbanization level

Medical cost had multicollinearity with length of days

Although urbanization did not precipitate the ROM, the patients cared for in local hospitals posed a higher ROM than those in regional hospitals or hospital centers (*P* = 0.006 and *P* = 0.032, respectively). Furthermore, the factors regarding LOS and medical costs did not increase the ROM after adjusting for variables.

### The changing trend of incidence rate among index patients

eFigure 2 shows the trend of the occurrence among index patients during the follow-up period. Overall, the incidence rate of index patients increased approximately tenfold from 0.14 per 105 population in 1998 to 1.46 per 105 population in 2015 (Table 3). Regarding sex, the incidence in female patients increased 8.5-fold from 0.18 per 105 population in 1998 to 1.54 per 105 population in 2015. Even though the incidence rate in male patients increased by 13.7-fold between 1998 and 2015, the changing trend of incidence rate was steadily higher in females than males during the follow-up period.

**Table 3:** Trend of the incidence rate of index patients

	**Overall**			**Male**			**Female**		
**Year**	**Inpatients**	**Mid-year population**	**Rate (per 10**^**5**^**)**	**Inpatients**	**Mid-year population**	**Rate (per 10**^**5**^**)**	**Inpatients**	**Mid-year population**	**Rate (per 10**^**5**^**)**
1998	30	21,928,591	0.14	11	11,243,408	0.10	19	10,685,183	0.18
1999	35	22,092,387	0.16	13	11,312,728	0.11	22	10,779,659	0.20
2000	58	22,276,672	0.26	20	11,392,050	0.18	38	10,884,622	0.35
2001	67	22,405,568	0.30	27	11,441,651	0.24	40	10,963,917	0.36
2002	92	22,520,776	0.41	38	11,485,409	0.33	54	11,035,367	0.49
2003	102	22,604,550	0.45	42	11,515,062	0.36	60	11,089,488	0.54
2004	118	22,689,122	0.52	45	11,541,585	0.39	73	11,147,537	0.65
2005	134	22,770,383	0.59	50	11,562,440	0.43	84	11,207,943	0.75
2006	158	22,876,527	0.69	66	11,591,707	0.57	92	11,284,820	0.82
2007	177	22,958,360	0.77	72	11,608,767	0.62	105	11,349,593	0.93
2008	198	23,037,031	0.86	86	11,626,351	0.74	112	11,410,680	0.98
2009	227	23,119,772	0.98	98	11,636,734	0.84	129	11,483,038	1.12
2010	240	23,162,123	1.04	109	11,635,225	0.94	131	11,526,898	1.14
2011	268	23,224,912	1.15	118	11,645,674	1.01	150	11,579,238	1.30
2012	279	23,315,822	1.20	121	11,673,319	1.04	158	11,642,503	1.36
2013	297	23,373,517	1.27	128	11,684,674	1.10	169	11,688,843	1.45
2014	318	23,433,753	1.36	142	11,697,971	1.21	176	11,735,782	1.50
2015	342	23,492,074	1.46	160	11,712,047	1.37	182	11,780,027	1.54
**Overall**	3,140	411,281,940	0.76	1,346	208,006,802	0.65	1,794	203,275,138	0.88

The annual percentage change (APC) was significantly noted in both sexes (male APC = 15.84, *P* < 0.001; female APC = 12.86, *P* < 0.001). Overall APC was 14.11, which also showed statistical significance (*P* < 0.001) (data not shown).

### The trend of the mortality rate and cut-off value of LOS

**Fig. 1:**
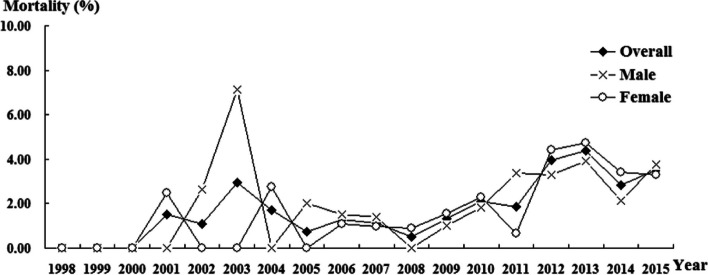
Trend of proportional mortality in index patients. Trend test (Joinpoint regression): Total: increase significantly, APC (Annual percentage change) = 7.79, *P* = 0.038. Male: increase slightly, APC = 157.32, *P* = 0.113. Female: increase significantly, APC = 239.34, *P* = 0.031

In males, the peak mortality rate of index patients was 7.14% in 2003, and mildly increased mortality was noted from 2008 to 2013 (eTable 3 and Fig. 1). In contrast, female patients had the highest mortality rate of 4.43% in 2012 and a steady mortality rate between 2012 and 2015. In addition, the trend of the overall mortality rate was similar to that of the female mortality rate.

**Fig. 2:**
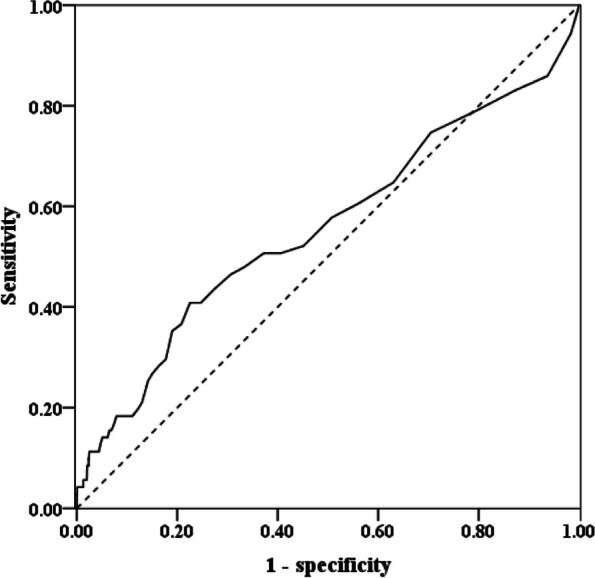
The optimal CoV of LOS to predict mortality by ROC curve. AUC (Area under the curve) = 0.658, 95% CI: 0.579—0.736, *P* = 0.026. The fitness cut-point of LOS: 16 days. CoV: cut-off value; LOS: length of hospital stay; ROC: receiver operating characteristic

Using the receiver operating characteristic (ROC) curve, we demonstrated that the optimal cut-off value (CoV) of LOS to predict the increased ROM was 16 days. The area under the ROC curves were 0.658 (95% CI: 0.579–0.736; *P* = 0.026) (eTable 4 and Fig. 2). In addition, index patients with long hospital LOS of > 16 days showed 2.3-fold increased ROM (≤ 16 days: 1.74%; > 16 days: 4.02%) (eTable 5).

## Discussion

### Summary of important results

To our knowledge, this is the first nationwide population-based study investigating the epidemiology of coexisting hypothyroidism and hyponatremia. Our results showed that < 1% of hyponatremia in patients was caused by hypothyroidism, and the average incidence was < 200 index patients per year, echoing the notion of rare coexistence. Females and those aged > 55 years accounted for the majority of index patients. Besides old age, clinical parameters such as underlying catastrophic illness, cardiac dysrhythmia, and treatment at a local hospital increased the ROM. Notably, the optimal CoV of LOS to predict the high ROM was first elucidated in this study, and the hospital LOS of > 16 days increased the ROM by 2.3-fold. Despite uncommon concurrence, the overall incidence rate of index patients increased approximately tenfold within 18 years. In accordance with thyroid dysfunction being common in women [16], the changing trend of incidence rate was steadily higher in females than males. The trend of the overall mortality rate was also similar to that of the female mortality rate. Taken together, our study not only increases the knowledge of epidemiology between hypothyroidism and hyponatremia but also clarifies the ROM parameters in these two concomitant diseases.

### Rare concurrence and low annual incidence

It has been reported that the frequency of hyponatremia was not different between adults with hypothyroidism and euthyroid controls [17] as well as between infants with congenital hypothyroidism and age-matched controls [18]. Recently, Krebs et al. reported that the prevalence of hyponatremia in patients with hypothyroidism was only 5.58%, and the association between thyroid function and serum sodium (Na^+^) concentrations was very weakly positive [16], suggesting that patients with hypothyroidism having moderate to severe hyponatremia often exhibited other underlying diseases concerning low Na^+^ values. Likewise, our large computerized database also confirmed this rarity of concurrence. In contrast, hypothyroidism-induced hyponatremia may be attributed to multiple mechanisms such as the combination of compartmental redistribution and water retention dilution of serum electrolytes (eg, increased ADH levels) [19] as well as a chronic dietary salt deficit [20]. Moreover, assessment of hyponatremia relies on estimating fluid volume, and this might be problematic because differentiating hypovolemia from euvolemia could sometimes be difficult in clinical practice [21]. Due to these constraints, the estimated prevalence of hyponatremia varies from 4 to 35%, given the diverse study designs [22, 23]. Even though the aforementioned difficulty led to the poor unraveling of the precise prevalence and the pathogenesis of hypothyroidism underpinning hyponatremia, our study took advantage of a large-scale study population to reduce recall and selection bias, demonstrating the uncommon concomitance and low annual incidence.

### Predictors of ROM in index patients

Despite the infrequent concurrence, hyponatremia per se is the most common electrolyte disorder for acute or chronic illnesses, commonly affecting older individuals [24, 25]. Furthermore, hyponatremia is associated with increased mortality and adverse outcomes, and it may also be deteriorated by hypothyroidism [20, 22]. Nevertheless, studies investigating the parameters of ROM among patients with the coexistence of hyponatremia and hypothyroidism are limited, especially in Asia. From this viewpoint, our results constructively show that the factors accentuating ROM in these patients include old age, catastrophic illness, cardiac dysrhythmia, and medical management in low hierarchical hospitals. Moreover, arrhythmia rather than HTN is an important factor of ROM. In support of this finding, Yilmaz et al. have reported that hyponatremia is independently associated with the occurrence of atrial fibrillation [26], which has a predilection for cardiac death.

### Increased overall annual incidence with female predominance

A previous study revealed that no sex predilection exists for hyponatremia, but hyponatremic symptoms are more likely to occur in young women [27]. In addition, thyroid disease is 10 times more common in females than in males [28, 29]. In accordance with the thyroid disorder, our study showed that the female index patients had a higher incidence rate than male patients, and the trend of the overall mortality rate was similar to that of the female mortality rate. Furthermore, the number of index patients became obvious since 2009, which might imply the increased recognition of concurrent hypothyroidism and hyponatremia [3, 16, 30–32]. Wolf et al. reported that co-occurrence of hyponatremia and hypothyroidism was not likely to be causal because, in patients with serum Na^+^ levels < 130 mmol/L, hyponatremia could not be attributed to impaired thyroid function [16]. In contrast, a retrospective cross-sectional study showed that the prevalence of overt hypothyroidism was significantly higher as the severity of hyponatremia progressed [31]. Although hypothyroidism has historically been implicated in the development of hyponatremia [33], this paradigm has been challenged, and it has been suggested that the link might merely be an association [30, 34]. Although our results showed an increased trend of annual incidence and the propensity for female patients when two diseases coexisted, the fact of < 200 index patients per year might also indicate that the causality was less likely. However, a large-scale prospective study is still necessary to clarify the issue as to whether this is a true causal relationship or mere coincidence.

### Strengths and limitations

This study had certain limitations. Firstly, socioeconomic (eg, educational level, occupation) and environmental factors, as well as biochemistry and endocrine parameters (eg, serum electrolyte, thyroid hormones/antibodies, and ADH), were not available in the NHIRD [35], thus it was unable to evaluate the severity of hypothyroidism or hyponatremia according to serum sodium, T3, T4, free T4, and thyroid-stimulating hormone levels. Secondly, this retrospective study lacked information regarding imaging and medical management during hospitalization, which may interfere with the analysis of mortality outcomes. Thirdly, some medical history associated with potential alternative causes and superimposed factors of hyponatremia, such as side effects of medication, concomitant underlying disease (proximal tubular dysfunction), or other endocrine disorders (adrenal insufficiency), were lacking in the present study [16]. Despite these limitations, there is probably complete ascertainment of the diagnoses of hypothyroidism and hyponatremia using the highly representative computerized data file for each individual from the NHIRD (comprehensive population coverage), resulting in less possibility of recall and selection bias [13]. 

## Conclusions

Hypothyroidism or hyponatremia per se is a common disease; however, their concurrence is rare, despite increasing annual incidence recently. In clinical care, factors such as old age, underlying catastrophic status, cardiac arrhythmia, low hospital hierarchy, and longer LOS significantly increased the ROM among the index patients. Our study findings have implications in improving the quality of care in geriatric patients. Early recognition of mortality predictors and optimal management of hypothyroidism behind severe hyponatremia is pivotal to prevent subsequent deleterious consequences of hyponatremia.

### Supplementary Information

**Additional file 1: eFigure 1.** Flowchart for study patient selection from the NHIRD. **eFigure 2.** Trend of the incidence rate in index patients. **eTable 1.** Abbreviation and ICD-9-CM. **eTable 2.** Proportional of unspecified acquired hypothyroidism among hyposmolality/hyponatremia.**eTable 3.** Trend of proportional mortality in index patients. **eTable 4.** Sensitivity and specificity of ROC curve of LOS. **eTable 5.** The optimal CoV of LOS to predict mortality by ROC curve.

## Data Availability

Data collected for the study and presented herein will be made available to others. Data will be organised in a data dictionary, and participant data will be de-identified. Related study documents, including the study protocol, and statistical analysis plan, will also be available. Data requests should be sent by email to the corresponding author (ming.sandra@msa.hinet.net).
