# Supplementary material for: An enigma of hypothyroidism and hyponatremia coexistence: a nationwide population-based retrospective study
Source: BMC Public Health. 2023 Sep 29;23:1889. doi: 10.1186/s12889-023-16791-5 (PMC10541708; doi:10.1186/s12889-023-16791-5)
Supplement: Supplementary file 1 — Additional file 1: eFigure 1. Flowchart for study patient selection from the NHIRD. eFigure 2. Trend of the incidence rate in index patients. eTable 1. Abbreviation and ICD-9-CM. eTable 2. Proportional of unspecified acquired hypothyroidism among hyposmolality/hyponatremia.eTable 3. Trend of proportional mortality in index patients. eTable 4. Sensitivity and specificity of ROC curve of LOS. eTable 5. The optimal CoV of LOS to predict mortality by ROC curve. [file 12889_2023_16791_MOESM1_ESM.docx]

# Supplementary information

# Figure legends

**eFigure 1.** **Flowchart for study patient selection from the NHIRD**

Unspecified acquired hypothyroidism: ICD-9-CM 244.9; Hyposmolality and /or hyponatremia: ICD-9-CM 276.1; Exclusion criteria: Sex or age unknown.

NHIRD, National Health Insurance Research Database.


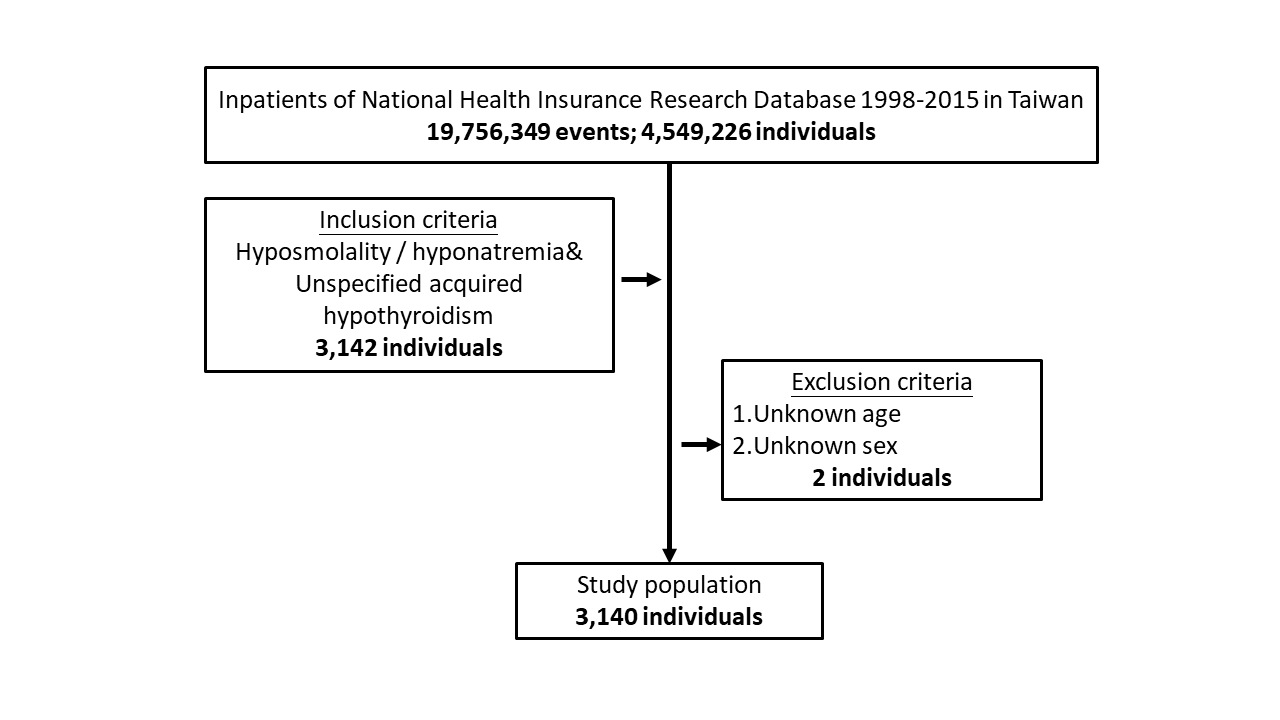


**eFigure 2. Trend of the incidence rate in index patients**

Trend test (Joinpoint regression):

Total: increase significantly, APC (Annual percentage change) = 14.11, *P* < .001

Male: increase significantly, APC = 15.84, *P* < .001

Female: increase significantly, APC = 12.86, *P* < .001

**
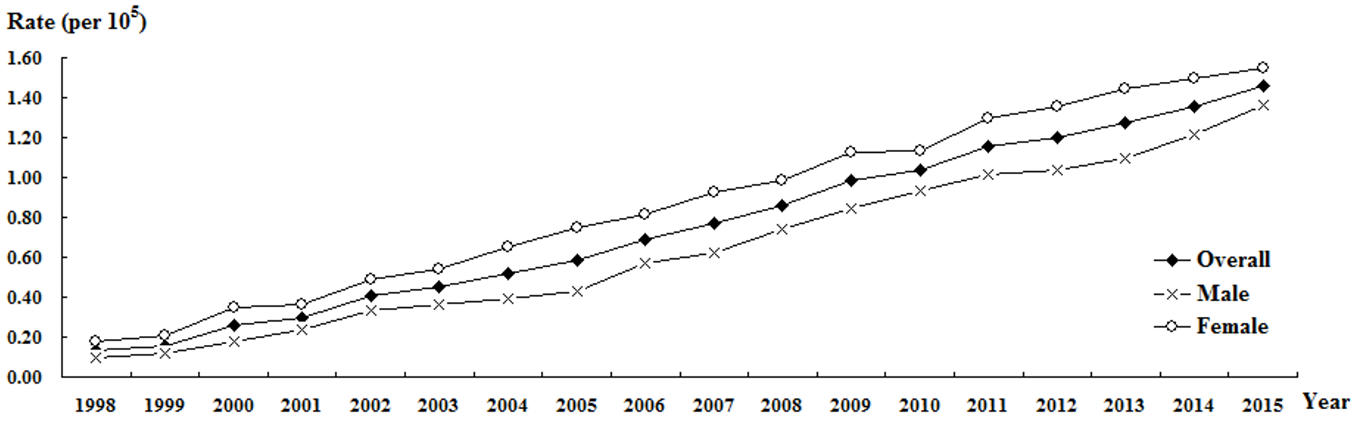
**

# Supplementary information

| **eTable 1. Abbreviation and ICD-9-CM** | | |
| --- | --- | --- |
|  | **Abbreviation** | **ICD-9-CM** |
| **Study population** |  |  |
| Hyposmolality / hyponatremia |  | 276.1 |
| Unspecified acquired hypothyroidism |  | 244.9 |
| **Comorbidities** |  |  |
| Diabetes mellitus | DM | 250 |
| Disorders of adrenal glands |  | 255 |
| Endocrine dysfunction |  | 258.1 |
| Hypertension | HTN | 401-405 |
| Ischemic heart disease | IHD | 410-414 |
| Heart failure | HF | 428 |
| Chronic kidney disease | CKD | 585 |
| Chronic obstructive pulmonary disease | COPD | 490-496 |
| Asthma |  | 493 |
| Mental disorders |  | 290-319 |
| Diseases of the nervous system |  | 320-389 |
| Cardiac dysrhythmias |  | 427 |
| Benign prostatic hyperplasia | BPH | 600.00 |
| Cardiomyopathy |  | 425 |
| Sickle-cell disease |  | 282.60 |
| Liver cirrhosis |  | 571 |
| Operations on bone marrow and spleen |  | OP41 |

| **eTable 2. Proportional of unspecified acquired hypothyroidism among hyposmolality/hyponatremia** | | |
| --- | --- | --- |
| **Unspecified acquired hypothyroidism** | n | % |
| Without | 350,992 | 99.11 |
| With | 3,142 | 0.89 |
| Overall | 354,134 |  |

| **eTable 3. Trend of proportional mortality in index patients** | | | | | | | | | |
| --- | --- | --- | --- | --- | --- | --- | --- | --- | --- |
|  | **Overall** | | | **Male** | | | **Female** | | |
| **Year** | **Mortality** | **Inpatients** | **Mortality %** | **Mortality** | **Inpatients** | **Mortality %** | **Mortality** | **Inpatients** | **Mortality %** |
| 1998 | 0 | 30 | 0.00 | 0 | 11 | 0.00 | 0 | 19 | 0.00 |
| 1999 | 0 | 35 | 0.00 | 0 | 13 | 0.00 | 0 | 22 | 0.00 |
| 2000 | 0 | 58 | 0.00 | 0 | 20 | 0.00 | 0 | 38 | 0.00 |
| 2001 | 1 | 67 | 1.49 | 0 | 27 | 0.00 | 1 | 40 | 2.50 |
| 2002 | 1 | 92 | 1.09 | 1 | 38 | 2.63 | 0 | 54 | 0.00 |
| 2003 | 3 | 102 | 2.94 | 3 | 42 | 7.14 | 0 | 60 | 0.00 |
| 2004 | 2 | 118 | 1.69 | 0 | 45 | 0.00 | 2 | 73 | 2.74 |
| 2005 | 1 | 134 | 0.75 | 1 | 50 | 2.00 | 0 | 84 | 0.00 |
| 2006 | 2 | 158 | 1.27 | 1 | 66 | 1.52 | 1 | 92 | 1.09 |
| 2007 | 2 | 177 | 1.13 | 1 | 72 | 1.39 | 1 | 105 | 0.95 |
| 2008 | 1 | 198 | 0.51 | 0 | 86 | 0.00 | 1 | 112 | 0.89 |
| 2009 | 3 | 227 | 1.32 | 1 | 98 | 1.02 | 2 | 129 | 1.55 |
| 2010 | 5 | 240 | 2.08 | 2 | 109 | 1.83 | 3 | 131 | 2.29 |
| 2011 | 5 | 268 | 1.87 | 4 | 118 | 3.39 | 1 | 150 | 0.67 |
| 2012 | 11 | 279 | 3.94 | 4 | 121 | 3.31 | 7 | 158 | 4.43 |
| 2013 | 13 | 297 | 4.38 | 5 | 128 | 3.91 | 8 | 169 | 4.73 |
| 2014 | 9 | 318 | 2.83 | 3 | 142 | 2.11 | 6 | 176 | 3.41 |
| 2015 | 12 | 342 | 3.51 | 6 | 160 | 3.75 | 6 | 182 | 3.30 |
| **Overall** | 71 | 3,140 | 2.26 | 32 | 1,346 | 2.38 | 39 | 1,794 | 2.17 |

| **eTable 4. Sensitivity and specificity of ROC curve of LOS** | | | |
| --- | --- | --- | --- |
| **Length of days** | **Sensitivity** | **1 - specificity** | **Sensitivity + Specificity** |
| 0 | 1.000 | 1.000 | 1.000 |
| 1 | 0.944 | 0.983 | 0.960 |
| 2 | 0.859 | 0.937 | 0.922 |
| 3 | 0.831 | 0.873 | 0.958 |
| 4 | 0.789 | 0.792 | 0.997 |
| 5 | 0.746 | 0.704 | 1.042 |
| 6 | 0.648 | 0.631 | 1.017 |
| 7 | 0.606 | 0.561 | 1.045 |
| 8 | 0.577 | 0.507 | 1.070 |
| 9 | 0.521 | 0.451 | 1.070 |
| 10 | 0.507 | 0.407 | 1.100 |
| 11 | 0.507 | 0.373 | 1.134 |
| 12 | 0.479 | 0.333 | 1.146 |
| 13 | 0.465 | 0.307 | 1.158 |
| 14 | 0.437 | 0.275 | 1.161 |
| 15 | 0.408 | 0.247 | 1.161 |
| **16** | **0.408** | **0.225** | **1.183** |
| 17 | 0.366 | 0.209 | 1.158 |
| 18 | 0.352 | 0.190 | 1.162 |
| 19 | 0.296 | 0.177 | 1.119 |
| 20 | 0.282 | 0.162 | 1.119 |
| 21 | 0.268 | 0.151 | 1.117 |
| 22 | 0.254 | 0.142 | 1.111 |
| 23 | 0.211 | 0.130 | 1.081 |
| 24 | 0.197 | 0.122 | 1.075 |
| 25 | 0.183 | 0.111 | 1.072 |
| 26 | 0.183 | 0.103 | 1.080 |
| 27 | 0.183 | 0.094 | 1.089 |
| 28 | 0.183 | 0.086 | 1.097 |
| 29 | 0.183 | 0.079 | 1.104 |
| 30 | 0.169 | 0.075 | 1.094 |
| 31 | 0.155 | 0.069 | 1.086 |
| 32 | 0.155 | 0.065 | 1.090 |
| 33 | 0.141 | 0.061 | 1.080 |
| 34 | 0.141 | 0.057 | 1.084 |
| 35 | 0.141 | 0.054 | 1.086 |
| 36 | 0.141 | 0.051 | 1.090 |
| 37 | 0.127 | 0.047 | 1.080 |
| 38 | 0.113 | 0.044 | 1.069 |
| 39 | 0.113 | 0.042 | 1.071 |
| 40 | 0.113 | 0.039 | 1.073 |
| 41 | 0.113 | 0.038 | 1.075 |
| 42 | 0.113 | 0.034 | 1.078 |
| 43 | 0.113 | 0.031 | 1.081 |
| 44 | 0.113 | 0.031 | 1.082 |
| 45 | 0.113 | 0.029 | 1.084 |
| 46 | 0.113 | 0.026 | 1.086 |
| 47 | 0.113 | 0.026 | 1.087 |
| 48 | 0.113 | 0.025 | 1.088 |
| 49 | 0.099 | 0.024 | 1.074 |
| 50 | 0.099 | 0.024 | 1.075 |
| 51 | 0.085 | 0.024 | 1.061 |
| 52 | 0.085 | 0.023 | 1.061 |
| 54 | 0.085 | 0.022 | 1.062 |
| 56 | 0.085 | 0.022 | 1.062 |
| 57 | 0.085 | 0.022 | 1.063 |
| 58 | 0.085 | 0.021 | 1.063 |
| 59 | 0.085 | 0.021 | 1.064 |
| 60 | 0.056 | 0.020 | 1.036 |
| 61 | 0.056 | 0.017 | 1.040 |
| 62 | 0.056 | 0.014 | 1.042 |
| 63 | 0.056 | 0.013 | 1.043 |
| 64 | 0.042 | 0.013 | 1.029 |
| 65 | 0.042 | 0.013 | 1.030 |
| 66 | 0.042 | 0.012 | 1.030 |
| 67 | 0.042 | 0.012 | 1.031 |
| 68 | 0.042 | 0.011 | 1.031 |
| 70 | 0.042 | 0.010 | 1.032 |
| 71 | 0.042 | 0.009 | 1.033 |
| 72 | 0.042 | 0.008 | 1.034 |
| 75 | 0.042 | 0.008 | 1.034 |
| 79 | 0.042 | 0.007 | 1.035 |
| 83 | 0.042 | 0.007 | 1.035 |
| 85 | 0.042 | 0.007 | 1.036 |
| 87 | 0.042 | 0.006 | 1.036 |
| 89 | 0.042 | 0.006 | 1.036 |
| 91 | 0.042 | 0.006 | 1.037 |
| 93 | 0.042 | 0.005 | 1.037 |
| 95 | 0.042 | 0.005 | 1.037 |
| 96 | 0.042 | 0.005 | 1.038 |
| 98 | 0.042 | 0.004 | 1.038 |
| 102 | 0.042 | 0.004 | 1.038 |
| 106 | 0.042 | 0.004 | 1.039 |
| 109 | 0.042 | 0.003 | 1.039 |
| 115 | 0.042 | 0.003 | 1.039 |
| 121 | 0.042 | 0.003 | 1.040 |
| 129 | 0.042 | 0.002 | 1.040 |
| 137 | 0.042 | 0.002 | 1.040 |
| 146 | 0.042 | 0.002 | 1.041 |
| 154 | 0.042 | 0.001 | 1.041 |
| 155 | 0.042 | 0.001 | 1.042 |
| 168 | 0.028 | 0.001 | 1.028 |
| 185 | 0.028 | 0.000 | 1.028 |
| 190 | 0.014 | 0.000 | 1.014 |
| 194 | 0.000 | 0.000 | 1.000 |
| 196 | 0.000 | 0.000 | 1.000 |

ROC: receiver operating characteristic; LOS: length of hospital stay

| **eTable 5. The optimal CoV of LOS to predict mortality by ROC curve** | | | | | | |
| --- | --- | --- | --- | --- | --- | --- |
| **Prognosis** | **Total** | | **Survive** | | **Mortality** | |
|  | n | % | n | % | n | % |
| **Length of days** | 3,140 |  | 3,069 | 97.74 | 71 | 2.26 |
| ≦16 | 2,419 | 77.04 | 2,377 | 98.26 | 42 | 1.74 |
| >16 | 721 | 22.96 | 692 | 95.98 | 29 | 4.02 |
| 1 | 55 | 1.75 | 51 | 92.73 | 4 | 7.27 |
| 2 | 149 | 4.75 | 143 | 95.97 | 6 | 4.03 |
| 3 | 197 | 6.27 | 195 | 98.98 | 2 | 1.02 |
| 4 | 252 | 8.03 | 249 | 98.81 | 3 | 1.19 |
| 5 | 272 | 8.66 | 269 | 98.90 | 3 | 1.10 |
| 6 | 233 | 7.42 | 226 | 97.00 | 7 | 3.00 |
| 7 | 218 | 6.94 | 215 | 98.62 | 3 | 1.38 |
| 8 | 166 | 5.29 | 164 | 98.80 | 2 | 1.20 |
| 9 | 176 | 5.61 | 172 | 97.73 | 4 | 2.27 |
| 10 | 136 | 4.33 | 135 | 99.26 | 1 | 0.74 |
| 11 | 106 | 3.38 | 106 | 100.00 | 0 | 0.00 |
| 12 | 124 | 3.95 | 122 | 98.39 | 2 | 1.61 |
| 13 | 81 | 2.58 | 80 | 98.77 | 1 | 1.23 |
| 14 | 99 | 3.15 | 97 | 97.98 | 2 | 2.02 |
| 15 | 88 | 2.80 | 86 | 97.73 | 2 | 2.27 |
| 16 | 67 | 2.13 | 67 | 100.00 | 0 | 0.00 |
| 17 | 55 | 1.75 | 52 | 94.55 | 3 | 5.45 |
| 18 | 58 | 1.85 | 57 | 98.28 | 1 | 1.72 |
| 19 | 43 | 1.37 | 39 | 90.70 | 4 | 9.30 |
| 20 | 47 | 1.50 | 46 | 97.87 | 1 | 2.13 |
| 21 | 37 | 1.18 | 36 | 97.30 | 1 | 2.70 |
| 22 | 27 | 0.86 | 26 | 96.30 | 1 | 3.70 |
| 23 | 39 | 1.24 | 36 | 92.31 | 3 | 7.69 |
| 24 | 27 | 0.86 | 26 | 96.30 | 1 | 3.70 |
| 25 | 35 | 1.11 | 34 | 97.14 | 1 | 2.86 |
| 26 | 25 | 0.80 | 25 | 100.00 | 0 | 0.00 |
| 27 | 25 | 0.80 | 25 | 100.00 | 0 | 0.00 |
| 28 | 26 | 0.83 | 26 | 100.00 | 0 | 0.00 |
| 29 | 21 | 0.67 | 21 | 100.00 | 0 | 0.00 |
| 30 | 15 | 0.48 | 14 | 93.33 | 1 | 6.67 |
| 31 | 18 | 0.57 | 17 | 94.44 | 1 | 5.56 |
| 32 | 12 | 0.38 | 12 | 100.00 | 0 | 0.00 |
| 33 | 13 | 0.41 | 12 | 92.31 | 1 | 7.69 |
| 34 | 13 | 0.41 | 13 | 100.00 | 0 | 0.00 |
| 35 | 8 | 0.25 | 8 | 100.00 | 0 | 0.00 |
| 36 | 11 | 0.35 | 11 | 100.00 | 0 | 0.00 |
| 37 | 13 | 0.41 | 12 | 92.31 | 1 | 7.69 |
| 38 | 10 | 0.32 | 9 | 90.00 | 1 | 10.00 |
| 39 | 6 | 0.19 | 6 | 100.00 | 0 | 0.00 |
| 40 | 8 | 0.25 | 8 | 100.00 | 0 | 0.00 |
| 41 | 5 | 0.16 | 5 | 100.00 | 0 | 0.00 |
| 42 | 11 | 0.35 | 11 | 100.00 | 0 | 0.00 |
| 43 | 9 | 0.29 | 9 | 100.00 | 0 | 0.00 |
| 44 | 2 | 0.06 | 2 | 100.00 | 0 | 0.00 |
| 45 | 5 | 0.16 | 5 | 100.00 | 0 | 0.00 |
| 46 | 8 | 0.25 | 8 | 100.00 | 0 | 0.00 |
| 47 | 1 | 0.03 | 1 | 100.00 | 0 | 0.00 |
| 48 | 3 | 0.10 | 3 | 100.00 | 0 | 0.00 |
| 49 | 4 | 0.13 | 3 | 75.00 | 1 | 25.00 |
| 50 | 1 | 0.03 | 1 | 100.00 | 0 | 0.00 |
| 51 | 1 | 0.03 | 0 | 0.00 | 1 | 100.00 |
| 52 | 2 | 0.06 | 2 | 100.00 | 0 | 0.00 |
| 53 | 2 | 0.06 | 2 | 100.00 | 0 | 0.00 |
| 55 | 1 | 0.03 | 1 | 100.00 | 0 | 0.00 |
| 56 | 1 | 0.03 | 1 | 100.00 | 0 | 0.00 |
| 57 | 2 | 0.06 | 2 | 100.00 | 0 | 0.00 |
| 58 | 2 | 0.06 | 2 | 100.00 | 0 | 0.00 |
| 59 | 4 | 0.13 | 2 | 50.00 | 2 | 50.00 |
| 60 | 10 | 0.32 | 10 | 100.00 | 0 | 0.00 |
| 61 | 8 | 0.25 | 8 | 100.00 | 0 | 0.00 |
| 62 | 3 | 0.10 | 3 | 100.00 | 0 | 0.00 |
| 63 | 1 | 0.03 | 0 | 0.00 | 1 | 100.00 |
| 64 | 1 | 0.03 | 1 | 100.00 | 0 | 0.00 |
| 65 | 2 | 0.06 | 2 | 100.00 | 0 | 0.00 |
| 66 | 1 | 0.03 | 1 | 100.00 | 0 | 0.00 |
| 67 | 2 | 0.06 | 2 | 100.00 | 0 | 0.00 |
| 69 | 3 | 0.10 | 3 | 100.00 | 0 | 0.00 |
| 71 | 2 | 0.06 | 2 | 100.00 | 0 | 0.00 |
| 72 | 3 | 0.10 | 3 | 100.00 | 0 | 0.00 |
| 73 | 1 | 0.03 | 1 | 100.00 | 0 | 0.00 |
| 77 | 2 | 0.06 | 2 | 100.00 | 0 | 0.00 |
| 82 | 1 | 0.03 | 1 | 100.00 | 0 | 0.00 |
| 84 | 2 | 200.00 | 2 | 100.00 | 0 | 0.00 |
| 85 | 1 | 0.03 | 1 | 100.00 | 0 | 0.00 |
| 88 | 1 | 0.03 | 1 | 100.00 | 0 | 0.00 |
| 90 | 1 | 0.03 | 1 | 100.00 | 0 | 0.00 |
| 91 | 1 | 0.03 | 1 | 100.00 | 0 | 0.00 |
| 94 | 1 | 0.03 | 1 | 100.00 | 0 | 0.00 |
| 95 | 1 | 0.03 | 1 | 100.00 | 0 | 0.00 |
| 96 | 1 | 0.03 | 1 | 100.00 | 0 | 0.00 |
| 100 | 1 | 0.03 | 1 | 100.00 | 0 | 0.00 |
| 103 | 1 | 0.03 | 1 | 100.00 | 0 | 0.00 |
| 108 | 1 | 0.03 | 1 | 100.00 | 0 | 0.00 |
| 109 | 1 | 0.03 | 1 | 100.00 | 0 | 0.00 |
| 120 | 1 | 0.03 | 1 | 100.00 | 0 | 0.00 |
| 122 | 1 | 0.03 | 1 | 100.00 | 0 | 0.00 |
| 135 | 1 | 100.00 | 1 | 100.00 | 0 | 0.00 |
| 139 | 1 | 0.03 | 1 | 100.00 | 0 | 0.00 |
| 153 | 2 | 0.06 | 2 | 100.00 | 0 | 0.00 |
| 154 | 1 | 0.03 | 1 | 100.00 | 0 | 0.00 |
| 155 | 1 | 0.03 | 0 | 0.00 | 1 | 100.00 |
| 181 | 1 | 0.03 | 1 | 100.00 | 0 | 0.00 |
| 188 | 1 | 0.03 | 0 | 0.00 | 1 | 100.00 |
| 192 | 1 | 0.03 | 0 | 0.00 | 1 | 100.00 |
| 195 | 1 | 0.03 | 1 | 100.00 | 0 | 0.00 |

CoV: cut-off value; ROC: receiver operating characteristic; LOS: length of hospital stay
